# Distribution characteristics of serum β2-microglobulin between viral and bacterial lower respiratory tract infections: a retrospective study

**DOI:** 10.7717/peerj.9814

**Published:** 2020-08-25

**Authors:** Xulong Cai, Qiaolan Xu, Chenrong Zhou, Li Zhou, Qijun Yong, Qing Mu, Yan Cheng, Jiena Wang, Jingjing Xie

**Affiliations:** Department of Pediatrics, Yancheng Third People’s Hospital, Yancheng, Jiangsu, China

**Keywords:** Serum β2-microglobulin, Lower respiratory tract, Viral infection

## Abstract

**Background:**

Lower respiratory tract infection (LRTI) is one of the leading cause of death in children under 5 years old around the world between 1980 and 2016. Distinguishing between viral and bacterial infection is challenging when children suffered from LRTI in the absence of pathogen detection. The aim of our study is to analyze the difference of serum β2-microglobulin (β2-MG) between viral LRTI and bacterial LRTI in children.

**Methods:**

This retrospective study included children with LRTI caused by a single pathogen from Yancheng Third People’s Hospital, Yancheng, China, between January 1, 2016 and December 31, 2019. Participants were divided into the younger group (1 year old ≤ age < 3 years old) and the older group (3 years old ≤ age < 5 years old) for subgroup analysis.

**Results:**

A total of 475 children with LRTI caused by common respiratory pathogens were identified. In the younger group as well as the older group, the serum level of β2-MG in respiratory syncytial virus, influenza A virus and influenza B virus groups were significantly increased compared to that in the *Mycoplasma pneumoniae* group. Compared with *Streptococcus pneumoniae* infection group, the serum β2-MG level of respiratory syncytial virus, influenza A virus and influenza B virus groups were significantly higher in children between 1 and 3 years old.

**Conclusions:**

The serum β2-MG may distinguish viral infection from bacterial infection in children with LRTI.

## Introduction

Lower respiratory tract infection (LRTI) is a common disease in children, and it also brings economic burden to families. The pathogenic microorganisms that caused LRTI mainly include viruses (respiratory syncytial virus, influenza virus, parainfluenza virus, adenovirus, coronavirus, human metapneumovirus, and rhinovirus) and bacteria (*Streptococcus pneumoniae*, *Haemophilus influenzae*, *Staphylococcus aureus*) (Musher & Thorner, 2014). LRTI is one of the leading cause of death in children under 5 years old around the world between 1980 and 2016 (GBD 2016 Causes of Death Collaborators, 2017). LRTI in children under 5 years old is mainly caused by virus (Tramper-Stranders, 2018). It was estimated that 56% of healthy children carry *Mycoplasma pneumoniae* (Meyer Sauteur et al., 2016). Both upper and lower respiratory tract infections can be induced by *M. pneumoniae*. School-age children and adolescents were the common population of *M. pneumoniae* infection (Waites et al., 2017). However, it has been observed that respiratory tract infections caused by *M. pneumoniae* are increasing among children younger than 5 years old (Sun et al., 2015). Elevated white blood cell is a useful parameter in the diagnosis of bacterial infections (Lavoignet et al., 2019). However, normal or decreased leukocyte counts do not exclude bacterial infection. Community acquired pneumonia caused by *M. pneumoniae* and virus have similar clinical symptoms, such as fever, muscle pain, weakness, dry cough. The white blood cell counts of viral infection is normal in most cases, as well as *M. pneumoniae* infection. It is difficult to distinguish *M. pneumoniae* from virus-induced LRTI (Sharma et al., 2017).

Rapid identification of pathogens is helpful for the treatment of LRTI. Early identification of viral infection can avoid overuse of antibiotics. The specificity of culture is high, but it takes a long time. The sensitivity of serological diagnosis depends on the time of sample collection (Waites et al., 2017). The polymerase-chain-reaction (PCR) detection technology improves the sensitivity of pathogen identification, but PCR is expensive (GBD 2016 Lower Respiratory Infections Collaborators, 2018). However, PCR is unable to identify all pathogens. Jain and colleagues used PCR technology to detect the pathogens in 2,259 community-acquired pneumonia patients, and 62% of the patients still could not identify the pathogens (Jain et al., 2015). Possible causes include unable to get lower respiratory tract specimen, application of antibiotics before specimen collection, insensitivity of diagnostic tests to known pathogens, lack of detection of other recognized pathogens, unidentified pathogens and noninfectious factors. Some biological indicators help to predict the pathogen categories of infectious diseases. Procalcitonin and C-reactive protein were considered to be biomarkers of bacterial infection in LRTI (Katz, Sartoni & Williams, 2019; Schuetz et al., 2017; IIrwin et al., 2017).

β2-MG is a nonglycosylated protein (11.6 kDa), which is found on the surface of almost all nucleated cells (Cunningham et al., 1973; Becker & Reeke, 1985). β2-MG is part of major histocompatibility complex class I that play a pivotal role in the adaptive immune system (Wieczorek et al., 2017). It was found that cytotoxic T cells were absent in β2-MG deficient mice (Zijlstra et al., 1990). Cytotoxic T cells activation occurs in viral infections (David et al., 2019). It has been found that abnormally high serum β2-microglobulin (β2-MG) levels are associated with several viral infections, such as human immunodeficiency virus, Epstein-Barr virus and cytomegalovirus (Zipeto et al., 2018; Lamelin et al., 1982; Norfolk, Barnard & Child, 1984).

Is there a better biomarker to identify viral infection in LRTI? We retrospectively studied the serum levels of β2-MG in patients with LRTI caused by respiratory syncytial virus, influenza A virus, influenza B virus *M. pneumonia* and *Streptococcus pneumonia*. In this study, the distribution difference of β2-MG in viral infection and bacterial infection was analyzed. We discussed the theoretical basis of β2-MG in distinguishing viral infection from bacterial infection. It was expected that the detection of β2-MG may provide help for the management of respiratory tract infection diseases, and antibiotic management.

## Methods

### Study design and participants

We reviewed the electronic medical records of all hospitalized children with a single pathogen of LRTI from Yancheng Third People’s Hospital, Yancheng, China, between January 1, 2016 and December 31, 2019. The LRTI in this study consisted of acute bronchitis and community-acquired pneumonia. The diagnosis of LRTI is based on the following criteria: diagnosis of acute bronchitis according to persistent cough less than 3 weeks and lack of radiologically visible infiltrates in lungs or other underlying lung disease. Diagnosis of community-acquired pneumonia according to fever, lung radiologically visible infiltrates, cough, sputum production, shortness of breath, abnormal breath sounds and rales in auscultation of lung. Pathogens include respiratory syncytial virus, influenza A virus, influenza B virus, *M. pneumonia* and *Streptococcus pneumonia*. Blood samples, sputum samples and nasopharynx swabs were collected and sent to the laboratory for pathogen detection within 24 h after admission.

Inclusion criteria: (1) Children suffered from LRTI. (2) There was only one pathogen causing LRTI. (3) Pathogens included *M. pneumoniae*, respiratory syncytial virus, influenza A virus, influenza B virus, *Streptococcus pneumoniae*. (4) β2-MG is often used as an index to evaluate renal function (Wong et al., 2016). The renal function of earlier infants is immature until the age of 8–12 months is close to that of adults (Kearns et al., 2003). Therefore, children were included in this study between 1 and 5 years old. The age of the child was based on the date of birth and the date of hospitalization. (5) Serum β2-MG had been detected in the acute stage of LRTI.

Exclusion criteria: (1) Children had primary immunodeficiency disease or kidney disease. (2) Immunodepressant was used in the last 2 weeks.

This study was approved by the ethics committee of the Yancheng Third People’s Hospital (Approval Number: 2019100). Individual informed consent was waived by the ethics committee of the Yancheng Third People’s Hospital because the study was considered to pose the least risk to participants.

### Pathogen detection

Viral infection: The diagnosis of respiratory syncytial virus, influenza A virus and influenza B virus infection were based on antigen detection or PCR nucleic acid detection of nasopharynx swab samples. *M. pneumoniae* infection: During hospitalization, the serum immunoglobulin M antibody of *M. pneumoniae* changed from negative to positive. *Streptococcus pneumoniae* infection: Sputum cultures or blood cultures were positive.

### Statistical analysis

Categorical variables were described by frequencies and percentage. Continuous variables features were expressed by mean ± standard deviation or interquartile ranges. The difference between the two groups of continuous variables is analyzed by student’s *t*-test. χ^2^-test was used to compare the difference of count data in different groups. Due to the different age distribution of different diseases, subgroup analysis of β2-MG was carried out according to age. All analysis were performed through SPSS 24 software.

## Results

### General characteristics of patients

**Figure 1:**
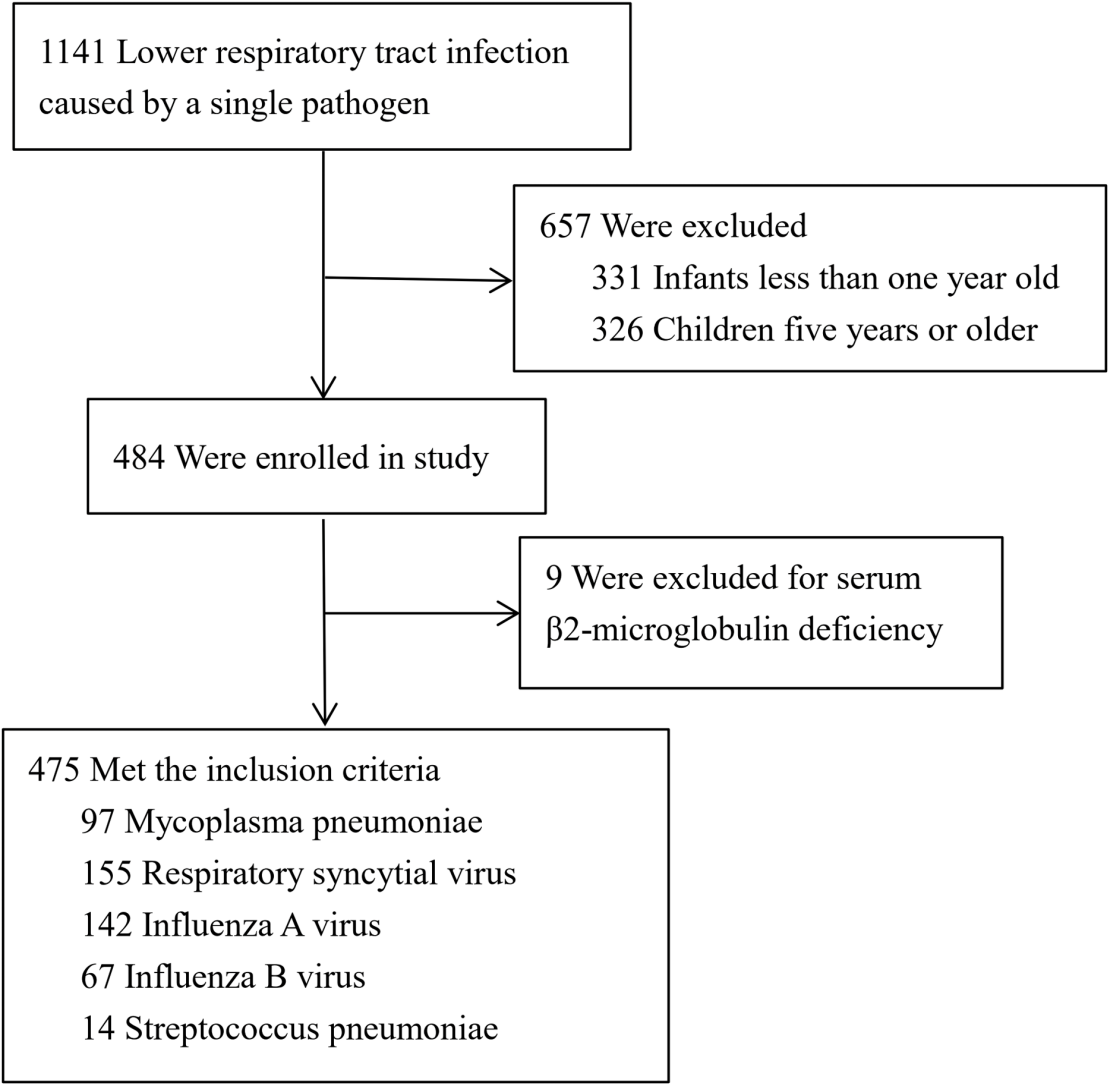
Screening of children with lower respiratory tract infection.

We collected 475 children with LRTI caused by single pathogen (Fig. 1). There were 155 cases of respiratory syncytial virus infection, 142 cases of influenza A virus infection, 67 cases of influenza B virus infection, 97 cases of *M. pneumoniae* infection and 14 cases of *Streptococcus pneumonia* infection. The epidemiological and clinical characteristics of patients were shown in Table 1. Blood urea nitrogen and serum creatinine were in normal range in all cases. Age distribution of children infected with different pathogens was analyzed. In the younger group (1 year old ≤ age < 3 years old), it is mainly 78.6% of children affected by respiratory syncytial virus, 52.1% of children affected by influenza A virus and 78.6% of children affected by *Streptococcus pneumonia*. In the older group (3 years old ≤ age < 5 years old), it is mainly 80.4% of children affected by *M. pneumoniae* and 62.7% of children affected by influenza B virus.

**Table 1:** Baseline characteristics of the patients.

Characteristic	MP	RSV	IAV	IBV	SP
	(*N* = 97)	(*N* = 155)	(*N* = 142)	(*N* = 67)	(*N* = 14)
Mean age ± SD, years	3.6 ± 1.0	2.3 ± 1.0	2.8 ± 1.1	3.3 ± 1.2	2.2 ± 1.2
1 ≤ age < 3, no. (%)	19 (19.6)	119 (76.8)	74 (52.1)	25 (37.3)	11 (78.6)
3 ≤ age < 5, no. (%)	78 (80.4)	36 (23.2)	68 (47.9)	42 (62.7)	3 (21.4)
Female, no. (%)	43 (44.3)	64 (41.3)	56 (39.4)	30 (44.8)	6 (42.9)
Wheezing, no. (%)	0	72 (46.5)	17 (12.0)	4 (6.0)	3 (21.4)
Febrile convulsion, no. (%)	0	5 (3.2)	15 (10.6)	5 (7.5)	1 (7.1)
WBC Median (IQR), ×10^9^/L	7.70 (6.29–9.87)	7.64 (5.84–10.47)	6.90 (5.22–8.92)	6.24 (4.25–8.86)	10.80 (7.28–13.27)
CRP ≥10 mg/L, no./total no. (%)^||^	39/95 (41.1)	23/154 (15.0)	25/141 (17.7)	8/66 (12.1)	5/14 (35.7)
BUN median (IQR), mmol/L	3.11 (2.48–3.62)	3.30 (2.59–4.05)	3.46 (2.76–4.22)	3.35 (2.71–4.09)	3.39 (2.92–3.60)
SCr median (IQR), μmol/L	31.4 (25.3–34.0)	28.0 (25.4–31.0)	33.0 (27.0–37.0)	33.0 (27.0–39.0)	27.5 (25.2–32.3)

**Notes:**

|| Cases with C-reactive protein test included in the statistics.

MP, *Mycoplasma pneumoniae*; RSV, Respiratory syncytial virus; IAV, Influenza A virus; IBV, Influenza B virus; SP, *Streptococcus pneumoniae*; WBC, white blood cell; CRP, C-reactive protein; IQR, quartile range; BUN, Blood urea nitrogen; SCr, serum creatinine; Normal reference intervals: BUN 1.43–8.20 μmol/L and SCr 22.0–132.6 μmol/L.

### Characteristics of β2-microglobulin in patients

Table 2 shows the characteristics of β2-MG in different pathogenic groups. In the younger group as well as the older group, the concentration of serum β2-MG in respiratory syncytial virus infection, influenza A virus infection and influenza B virus infection were significantly increased compared to that in *M. pneumoniae* infection. There were only three cases of lower respiratory tract infection with *Streptococcus pneumoniae* in children between 3 and 5 years old, so there was no statistical comparison. The concentration of serum β2-microglobulin was 1.67 mg/L, 1.69 mg/L and 1.51 mg/L, respectively. The β2-MG concentration in viral infection group was compared with that in *Streptococcus pneumoniae* infection group. The β2-MG concentration was significantly increased in respiratory syncytial virus group, influenza A virus group and influenza B virus group (Table 3). The serum β2-MG level was significantly different between the younger group and the older group in the LRTI caused by influenza A virus (Table 4).

**Table 2:** Serum β2-MG levels in children with *Mycoplasma pneumoniae* lower respiratory tract infection compared to those with viral lower respiratory tract infection.

Variable	*N*	Age		Sex		β2-MG		β2-MG > 2.8 mg/L	
		Mean ± SD (years)	*P* value	Female no. (%)	*P* value	Mean ± SD (mg/L)	*P* value	no./total no. (%)	*P* value
1 ≤ age < 3									
MP	19	2.0 ± 0.6	Ref.	7 (36.8)	Ref.	2.53 ± 0.31	Ref.	3/19 (15.8)	Ref.
RSV	119	1.9 ± 0.6	0.287	51 (42.9)	0.622	2.81 ± 0.60	0.003	64/119 (53.8)	0.002
IAV	74	1.9 ± 0.5	0.337	31 (41.9)	0.69	3.10 ± 0.66	<0.001	48/74 (64.9)	<0.001
IBV	25	2.0 ± 0.6	0.859	13 (52.0)	0.317	3.27 ± 0.96	<0.001	14/25 (56.0)	0.007
3 ≤ age < 5									
MP	78	4.0 ± 0.6	Ref.	36 (46.2)	Ref.	2.39 ± 0.37	Ref.	7/78 (9.0)	Ref.
RSV	36	3.9 ± 0.5	0.234	13 (33.3)	0.197	2.64 ± 0.44	0.002	13/36 (36.1)	<0.001
IAV	68	3.9 ± 0.6	0.229	25 (36.8)	0.251	2.85 ± 0.73	<0.001	33/68 (48.5)	<0.001
IBV	42	4.1 ± 0.6	0.644	17 (40.4)	0.55	2.99 ± 0.74	<0.001	28/42 (66.7)	<0.001

**Note:**

MP, *Mycoplasma pneumoniae*; RSV, Respiratory syncytial virus; IAV, Influenza A virus; IBV, Influenza B virus; SP, *Streptococcus pneumoniae*; Normal reference intervals, Serum β2-microglobulin (β2-MG) 0.8–2.8 mg/L.

**Table 3:** Serum β2-MG levels in children with *Streptococcus pneumoniae* lower respiratory tract infection compared to those with viral lower respiratory tract infection.

Variable	*N*	Age		Sex		Serum β2-MG	
		Mean ± SD (years)	*P* value	Female, no. (%)	*P* value	Mean ± SD (mg/L)	*P* value
1 ≤ age < 3							
SP	11	1.6 ± 0.6	Ref.	5 (45.5)	Ref.	2.24 ± 0.71	Ref.
RSV	119	1.9 ± 0.6	0.262	51 (42.9)	0.868	2.81 ± 0.60	0.003
IAV	74	1.9 ± 0.5	0.184	31 (41.9)	0.823	3.10 ± 0.66	<0.001
IBV	25	2.0 ± 0.6	0.144	13 (52.0)	0.717	3.27 ± 0.96	0.003

**Note:**

SP, *Streptococcus pneumoniae*; RSV, Respiratory syncytial virus; IAV, Influenza A virus; IBV, Influenza B virus; β2-microglobulin, β2-MG.

**Table 4:** Distribution characteristics of serum β2-MG in different age groups.

Variable	*N*	Mean ± SD (mg/L)	*P* value
MP			
1 ≤ age < 3	19	2.53 ± 0.31	0.137
3 ≤ age < 5	78	2.39 ± 0.37	
RSV			
1 ≤ age < 3	119	2.81 ± 0.60	0.117
3 ≤ age < 5	36	2.64 ± 0.44	
IAV			
1 ≤ age < 3	74	3.10 ± 0.66	0.034
3 ≤ age < 5	68	2.85 ± 0.73	
IBV			
1 ≤ age < 3	25	3.27 ± 0.96	0.181
3 ≤ age < 5	42	2.99 ± 0.74	

**Note:**

Age, year; MP, *Mycoplasma pneumoniae*; RSV, Respiratory syncytial virus; IAV, Influenza A virus; IBV, Influenza B virus.

## Discussion

In our research, children with LRTI caused by respiratory syncytial virus, influenza A virus and influenza B virus had higher levels of serum β2-MG than children with LRTI caused by *M. pneumoniae*. The results suggest that serum β2-MG may be helpful to distinguish *M. pneumoniae* infection from viral infection in LRTI. Tamura et al. (2008) found that urine β2-MG increased in children with refractory *M. pneumoniae* pneumonia. β2-MG freely passes through the glomerular filtration barrier. Urinary β2-MG increased when serum β2-MG level exceeded renal threshold. It was found that macrophages infected with influenza A virus could temporarily increase the expression of β2-MG (Keskinen et al., 1997). Our results suggest that the concentration of β2-MG increased significantly in children with viral LRTI.

We analyzed virus groups and *Streptococcus pneumoniae* group between 1 and 3 years old. The serum β2-MG level of respiratory syncytial virus group, influenza A virus group and influenza B virus group were higher than that of the *Streptococcus pneumoniae* group. Previous studies had found that the increased concentration of serum β2-MG was related to human cytomegalovirus, Epstein-Barr virus, hepatitis C virus and hepatitis B virus (Fabbri et al., 2011; Grywalska et al., 2015; Dlouhy et al., 2017; Deveci et al., 2020). The level of serum β2-MG was increased in patients with human immunodeficiency virus, and its concentration changes reflected the progress of the disease (Chitra, Bakthavatsalam & Palvannan, 2011). Overall, serum β2-MG may be more specific in the identification of viral infection.

Correlation analysis showed that the serum β2-MG level decreased with the increase of gestational age in normal fetuses (Hu et al., 2014). We analyzed the relationship between serum β2-MG level and age in children with LRTI. We only observed that serum β2-MG level was significantly reduced in older children with LRTI caused by influenza A virus. In the case of infection, whether the concentration of serum β2-MG was affected by age needs further study.

There are some obvious limitations in our study, and the results may be biased. First, this retrospective study collected a small number of samples. Second, although we had screened cases with a single pathogen infection, the limitations of clinical pathogen detection may be combined with other undetected pathogens. Third, the vast majority of the bacterial cases in this study were of *M. pneumoniae*, therefore the generalisability of these findings to other bacterial infections is limited. Fourth, we did not perform any multivariate linear regression models to see if viral/non-viral atiology was an independent predictor of higher β2-MG levels, after including possible confounders such as age, creatinine/urea nitrogen levels, gender, C-reactive protein, white blood cell. Fifth, we lack analysis of healthy children.

## Conclusions

In a retrospective analysis of the children with lower respiratory tract infection, we found that serum β2-MG levels in respiratory syncytial virus, influenza A virus, and influenza B virus groups were higher than *M. pneumoniae* group and *Streptococcus pneumoniae* group. Hence we infer that serum β2-MG may play an important role in the prediction of viral LRTI in children. It may provide a basis for avoiding antibiotic abuse. The molecular biological mechanism of β2-MG needs further verification and exploration.

## Supplemental Information

10.7717/peerj.9814/supp-1 Supplemental Information 1 Raw data.

## Additional Information and Declarations

### Data Availability

The following information was supplied regarding data availability: The raw measurements are available in the Supplemental Files.
